# Insights Into Subspecies Discrimination Potentiality From Bacteria MALDI-TOF Mass Spectra by Using Data Mining and Diversity Studies

**DOI:** 10.3389/fmicb.2020.01931

**Published:** 2020-08-13

**Authors:** Audrey Giraud-Gatineau, Gaetan Texier, Eric Garnotel, Didier Raoult, Hervé Chaudet

**Affiliations:** ^1^IHU Méditerranée Infection, Marseille, France; ^2^Assistance Publique Hôpitaux de Marseille, Marseille, France; ^3^Aix Marseille Univ., IRD, AP-HM, SSA, VITROME, IHU Méditerranée Infection, Marseille, France; ^4^Centre d’Epidémiologie et de Santé Publique des Armées (CESPA), Marseille, France; ^5^Hôpital d’Instruction des Armées Laveran, Marseille, France; ^6^Aix Marseille Univ., IRD, AP-HM, MEPHI, Marseille, France

**Keywords:** MALDI-TOF, proteomics, bacterial diversity, species subgrouping, pathogen spread, cluster analysis

## Abstract

Bacterial identification at subspecies level is critical in clinical care and epidemiological investigations due to the different epidemic potentialities of a species. For this purpose, matrix-assisted laser desorption ionization – time-of-flight mass spectrometry (MALDI-TOF MS) has been proposed in place of molecular genotyping, but with some result discrepancies. The aim of this work is to methodically mine the expression diversities of MALDI-TOF bacterial species spectra and their possible latent organization in order to evaluate their subspecies specific expression. Peak expression diversities of MALDI-TOF spectra coming from routine identifications have been analyzed using Hill numbers, rarefaction curves, and peak clustering. Some size effect critical thresholds were estimated using change point analyses. We included 167,528 spectra corresponding to 405 species. Species spectra diversities have a broad size-dependent variability, which may be influenced by the kind of sampling. Peak organization is characterized by the presence of a main cluster made of the most frequently co-occurring peaks and around 20 secondary clusters grouping less frequently co-occurring peaks. The 35 most represented species in our sample are distributed in two groups depending on the focusing of their protein synthesis activity on the main cluster or not. Our results may advocate some analogy with genomics studies of bacteria, with a main species-related cluster of co-occurring peaks and several secondary clusters, which may host peaks able to discriminate bacterial subgroups. This systematic study of the expression diversities of MALDI-TOF spectra shows that latent organization of co-occurring peaks supports subspecies discrimination and may explain why studies on MALDI-TOF-based typing exhibit some result divergences.

## Introduction

Bacterial identification at species level nowadays becomes less and less adequate for clinical care and epidemiological investigation (Herd and Kocks, 2001; Faruque et al., 2004; Freitas et al., 2016). Actually, a same species is a heterogeneous population gathering a diversity of strains that have different epidemic and pathogenic capabilities (Fournier et al., 2007; Sintchenko et al., 2007; Sabat et al., 2013; Sintchenko and Holmes, 2015). Strain characterization allows to find the origin of a bacterial clone, to follow its temporal and spatial spread and also to recognize highly virulent or/and resistant strains leading to change our diagnostic, therapeutic approach and outbreak management.

Knowledge about the strain composition of a bacterial species is granted by the study of genomes, sequencing partially or totally the DNA. Many methods have been used to tackle the diversity of a bacterial species: ribotyping (Bouchet et al., 2008), pulsed-field gel electrophoresis (PFGE; Heinzen et al., 1990), multilocus sequence typing (MLST; Maiden et al., 1998), polymerase chain reaction (PCR) with primers (Frazier et al., 1990), or whole genome sequencing (WGS; Kwong et al., 2015). But all these methods have a significant human and financial cost, restricting their systematic use. It is the reason why many studies tried to replace genotyping by the use of matrix-assisted laser desorption/ionization – time-of-flight mass spectrometry (MALDI-TOF MS) during epidemiological studies (Griffin et al., 2012; Berrazeg et al., 2013; Christner et al., 2014; Khennouchi et al., 2015) for deepening the bacterial identification at the strain level (Karas et al., 1985; Seng et al., 2009). The composition of MALDI-TOF spectra has been studied in the past and it has been demonstrated that peaks constituting spectra match with ribosomal proteins, DNA-binding proteins, cold shock proteins, and metabolites (Holland et al., 1999; Fenselau and Demirev, 2001; Ryzhov and Fenselau, 2001). But, as previous studies showed some discrepancies in MALDI-TOF MS capacity for discriminating subgroups within bacteria species, the ability of this technique for sub-species typing has been questioned (Sauget et al., 2017) and reviewed (Spinali et al., 2015).

For understanding the possible reasons of these discrepancies, and in the search for spectra compositions supporting species subgrouping, we have methodically analyzed, in a data-mining study, 4 years of routine standardized bacterial identifications extracted from our MALDI-TOF MS datawarehouse, and characterized by same growing conditions and sample preparations. The aim of this work is to analyze the expression diversities of spectra coming from 405 bacterial species. We successively present an overall analysis of spectra richness and the conditions affecting its variability, then a diversity study of peak-co-occurring in search of latent organization rules between species-related and sub-species-related peaks.

## Materials and Methods

### Raw Material

The Institut Hospitalo-Universitaire Mediterranée Infection (IHUMI) owns an asset of more than 4 millions of MALDI-TOF MS spectra produced since 2011, including 900,000 spectra of routine bacterial identifications for the four university hospitals of the Assistance Publique – Hôpitaux de Marseille and for the Hôpital d’instruction des armées Laveran in Marseille. The patient recruitment of these hospitals is mainly regional, from the South-East of France. All other spectra, which come from research activities and are related to microbiome studies, parasites, insects, environment, and diverse secretions, were excluded from this study.

All routine bacterial identifications are coming only from cultures growing on blood agar and chocolate agar, depending on the species. The culture is systematically stopped during the middle of the log phase, after a time lapse varying from 4 h to 10 days depending on the species growing speed. In this way, as bacteria are in rich and standardized medium, their biosynthesis is mainly used for the production of ribosomes and ribosome affiliated proteins, with a minimum of exogenous proteins and metabolic enzymes (Kim et al., 2020), and avoid condition-dependent protein abundance (Schmidt et al., 2016). As the spectra are expected to allow species identification by comparison against a reference database, cultures must be as standardized and as close as possible to the conditions used for the reference spectra. All spectra were done using three Bruker Microflex mass spectrometers following the standardized protocol provided by Bruker Daltonics. Single colony or sediment is directly applied on two distinct spots on ground steel targets, air dried, overlaid with 1 μl of a saturated α-cyano-4-hydroxycinnamic acid matrix solution in 50% of acetonitrile and 2.5% of trifluoroacetic acid, and air dried for 5 min. Spectra acquisitions are controlled using FlexControl software. Spectra were acquired using the spectrometer default settings (positive linear mode within the m/z range of 2–20 kDa, laser frequency 60 Hz; ion source 1 voltage, 20 kV; ion source 2 voltage, 16.7 kV; and lens voltage, 7.0 kV), and using 240 laser shots at 60 Hz in 40-shot steps from different locations. The Bruker Bacterial Test Standard (BTS; an extract of *Escherichia coli* DH5 alpha with two additional proteins, from Bruker Daltonics, Germany) for calibration was systematically used according to Bruker’s instructions. For each plate, BTS was used as a positive control and a non-inoculated-matrix solution as a negative control. Bacterial species identification is given using Biotyper software with an identification at species level when log (score) is ≥2.0. All MALDI-TOF MS spectra produced by the automatons were loaded in a data warehouse, along with the corresponding biological sample data (anonymized patient demographics and stay characteristics, sample characterization, requesting unit, species identification, and antibiogram).

### Inclusion Criteria

This study has been allowed by the French Data Protection Authority (CNIL decision DR-2018-177), and declared on ClinicalTrials.gov Protocol Registration and Result System (id: NCT03626987). We included in our study all spectra coming from routine clinical bacterial identification given at species level after bacterial culture, retaining samples with only one species found, and produced between February, 1st 2014 (beginning of the availability of associated biological data) and November, 30th 2018 (endpoint date). We excluded identifications producing more than two spectra for a same isolate, which could suggest some identification issues. A sample will then be represented by at most two spectra in this study. We also excluded bad quality spectra possibly due to anomalies of the amount of pellet with the method suggested by Palarea-Albaladejo et al. (2018). For computer processing capability reasons, the number of spectra for a species was limited to a maximum of 15,000, the representativeness being controlled by a random sampling over the study period.

### Spectra Processing

All selected spectra were processed for building a spectra peak database, using a homemade program written in R software (R core team, 2018) with MALDIquant package (Gibb and Strimmer, 2012). During the whole process, we used signal to noise ratio (SNR) = 2 as peak detection threshold and 300 ppm as peak deviation tolerance (corresponding to the built-in Microflex tolerance). We choose SNR = 2 for building an exhaustive peak inventory, giving the possibility of later increasing the selectivity with a more stringent SNR for some analyses.

During the first process step, the spectra are normalized as recommended by Gibb and Strimmer (2012): after intensity transformation (square root method), signal smoothing (moving average method, half-window size = 12), baseline removal (Statistics-sensitive Non-linear Iterative Peak-clipping algorithm, 100 iterations), spectra were normalized using total ion current method. All parameters of this step were chosen in accordance with the sensitivity study published by Christner et al. (2014).

During the second step, species spectra were successively aligned using cubic warping functions for correcting the machine drift and then for inter-spectra alignment. The drift-correcting warping function was determined for each target plate by using the eight reference peaks (3637.8, 5096.8, 5381.4, 6255.4, 7274.5, 10300.1, 13683.2, and 16952.3 Da) of the BTS required for each target plate. Spectra without plate control or with reference peaks out of the built-in Microflex tolerance window were dropped. The inter-spectra alignment warping function was determined for each species on all its spectra using peaks having a minimum occurrence frequency of 50%.

During the third step, we built two peak matrices: an intensity matrix and a SNR matrix. Along with these matrices, a table of descriptive statistics (including the numbering, mean, variance, and percentiles with a resolution of 5%) built for each peak present in the species spectra was stored in a dedicated database.

### Data Mining and Diversity Studies

Diversity analysis and data mining were done using R with Vegan (Oksanen et al., 2019), iNEXT (Hsieh et al., 2016), and dynamicTreeCut (Langfelder et al., 2008) packages. As mentioned above, we have raised the SNR threshold to three during these analyses for increasing the discriminating power of the analyses.

Diversity studies were based on Hill numbers (richness, exponential of Shannon entropy, and inverse of Simpson concentration), Chao’s richness estimator, and rarefaction curves (Jost, 2006; Chao et al., 2014).

Peak co-occurring study was done using cluster analysis (principal component analysis, K-means, and linear discriminant analysis). For each species, we computed the distance between two peaks as 1 minus the relative frequency of the peak co-occurring. We used the resulting distance matrix for an ascending hierarchical cluster analysis (weighted pair group method with arithmetic mean, WPGMA, for avoiding the absorption of little groups by the predominant ones) in search of species related groups of frequent co-occurring peaks. For detecting clusters, we used the dynamic hybrid cut method, which analyses the shape of the dendrogram branches in place of a static tree cut at a specific height (Langfelder et al., 2008). The description of this algorithm can be found at the following link: https://horvath.genetics.ucla.edu/html/CoexpressionNetwork/BranchCutting/. We used it with the following parameters: minimal cluster size of three peaks, cut height at 0.99, core scatter at 0.99, minimal gap of 0.007.

Change point of data series were found using Bai and Perron’s method for linear regression models (Bai and Perron, 1998).

**Figure 1:**
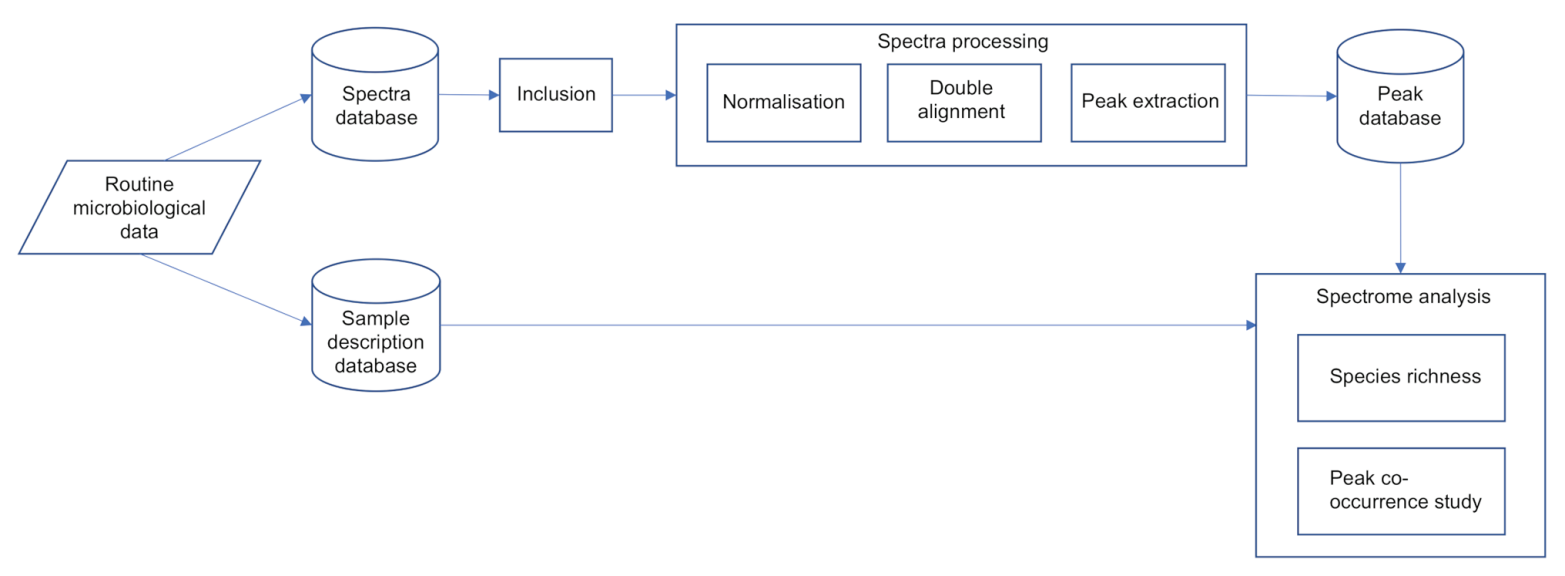
The overall spectra process flow.

The overall process flow is described in Figure 1.

## Results

### Inclusions

**Figure 2:**
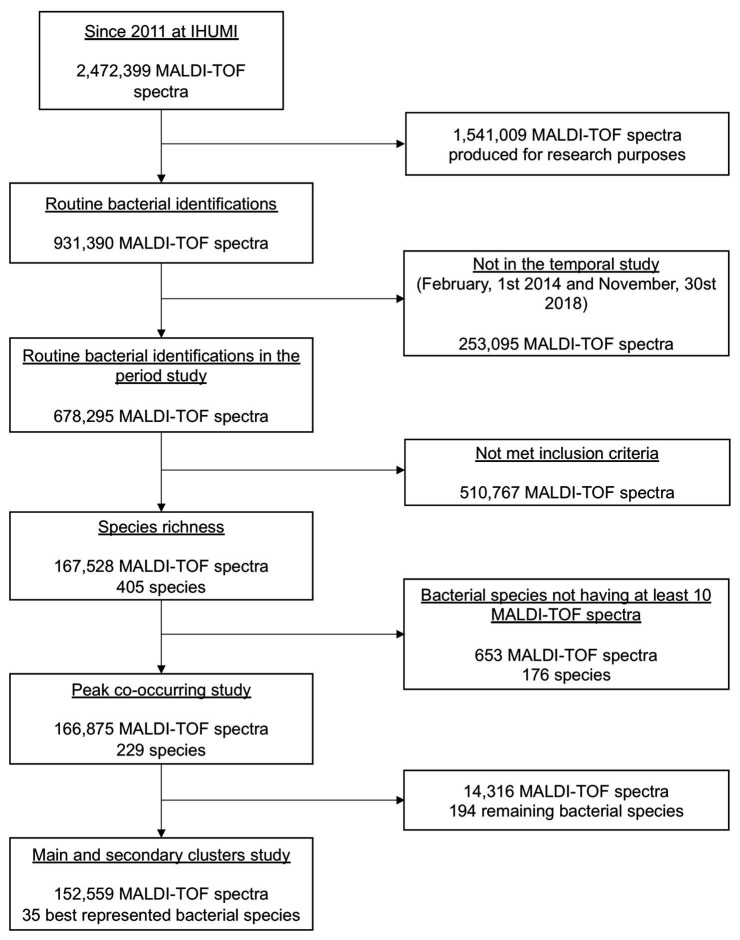
The flow diagram of the study.

During our study period, 2,49,075 bacterial identifications were performed at the IHUMI (2,13,436 clinical samples, 83,555 patients, and 1,15,614 stays), corresponding to 6,78,295 MALDI-TOF MS spectra. Only 1,67,528 spectra and 405 species met the inclusion criteria. The inclusion flow chart of this study is presented in Figure 2.

The double alignment process quality was verified using the *Es. coli* spectra set and the 6 species peaks known to be systematically present (proteins RL29 [M + 2H]^2+^, RS32 [M + H]^+^, RS34 [M + H]^+^, RS33 meth [M + H]^+^, RL29 [M + H]^+^, and RS19 [M + H]^+^), and the deviations ranged from 16 to 220 ppm, within the 300 ppm tolerance limits.

### Species Richness

**Figure 3:**
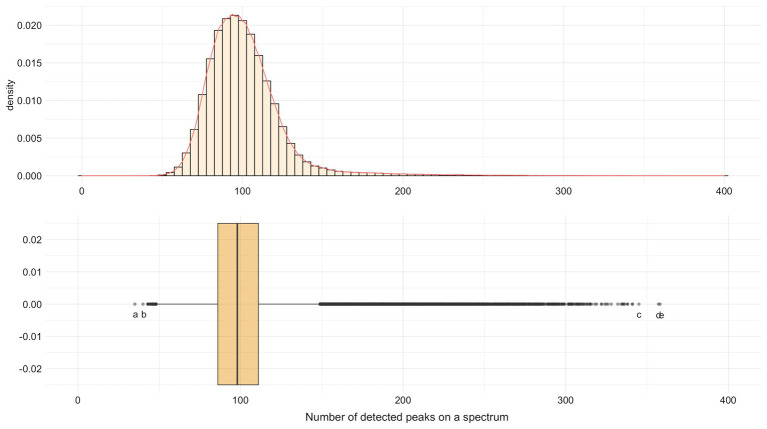
Distribution of the number of detected MALDI-TOF MS peaks by spectrum for SNR ≥ 2. Extreme values: ^a^ *Enterococcus faecalis* with 35 peaks. ^b^ *Pseudomonas aeruginosa* with 40 peaks. ^c^ *Staphylococcus pasteuri* with 345 peaks. ^d^ *Staphylococcus epidermidis* with 357 peaks. ^e^ *Klebsiella oxytoca* with 358 peaks.

In average, 101.02 peaks/spectrum (*SD* = 24.21, median = 98, min = 35, max = 358) were found. The minimum (*N* = 35) was for a spectrum of *Enterococcus faecalis* and the maximum (*N* = 358) for a spectrum of *Klebsiella oxytoca* (Figure 3). Indeed, 96.8% (*N* = 1,62,203) of spectra had between 50 and 150 detected peaks.

In the following, we will call “panspectrome” the set of all MALDI-TOF MS peaks retrieved for a bacterial species.

**Figure 4:**
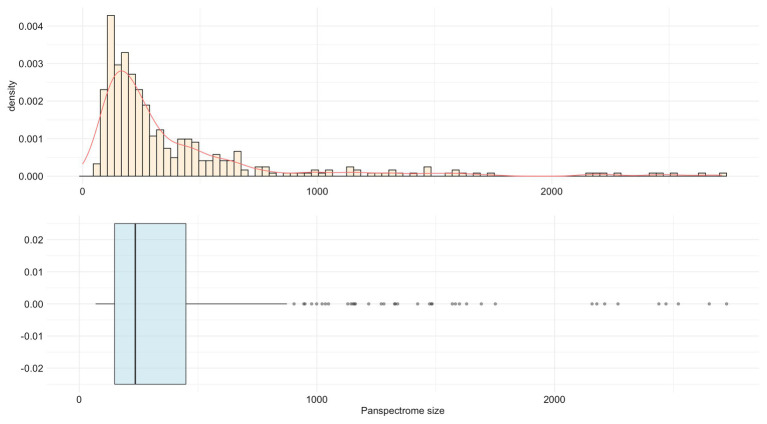
Distribution of the number of species for each panspectrome size for the 405 studied bacterial species.

*Staphylococcus epidermidis* presented the largest panspectrome (2,724 peaks retrieved for this species with SNR ≥ 2 and 1,788 peaks with SNR ≥ 3). Inversely, *Bordetella petrii* had the smallest panspectrome (69 peaks with SNR ≥ 2 and 55 peaks with SNR ≥ 3; Supplementary Table S1). The distribution of the number of species for each panspectrome size is reported in Figure 4.

**Figure 5:**
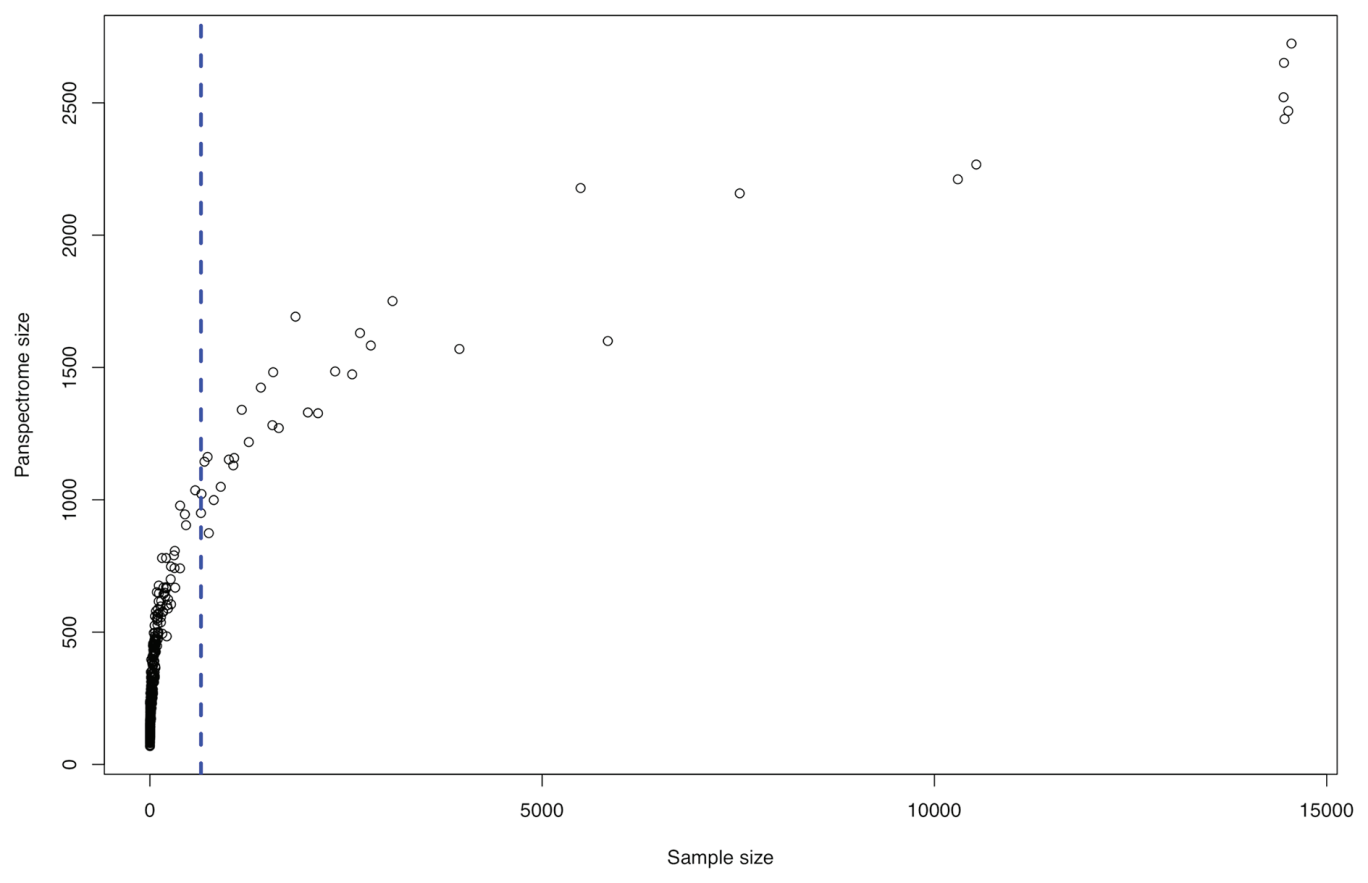
Spectrome size related to the sample size for the 405 bacterial species.

The panspectrome richness was conditioned by the number of spectra by species (Figure 5), which is itself function of the number of clinical cases (Supplementary Table S1). For example, *Enterobacter cloacae* gave a panspectrome richness of 2,267 peaks but for 10,533 spectra, while *Acinetobacter baumannii* gave a panspectrome of no more than 1,144 peaks for only 695 spectra. Despite the apparent important constraint of the sample size, the analysis of the accumulation curves using Chao’s extrapolation method (Chao et al., 2014) showed that a median of 365 spectra (first Qu. = 272 and third Qu. = 592) allows a sample coverage of 99%. This means that the acquisition cost of 99% of the panspectrome for 75% of the species is around 600 spectra.

**Figure 6:**
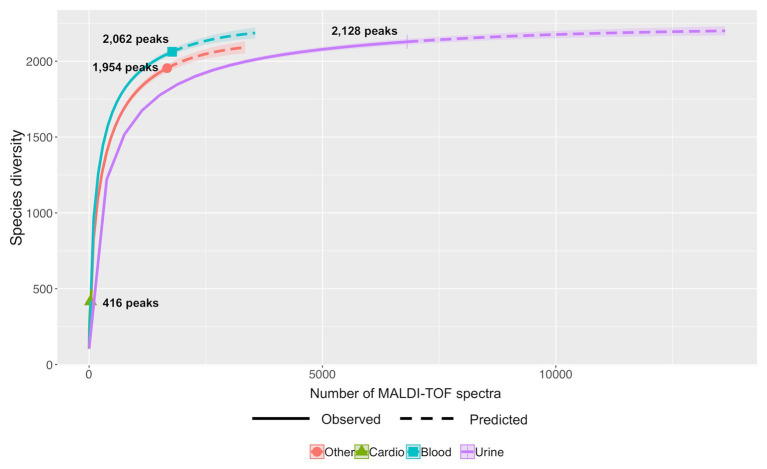
Predictive accumulation curves for *En. faecalis* according to the kind of samples: urine, blood, cardiac, or other.

Differential studies of Hill numbers and accumulation curves showed also that the richness of a same bacterial species varies with the kind of sampling. For example (Figure 6), *En. faecalis* showed a higher diversity for strains from blood cultures (Shannon index of 344.31 and Simpson diversity of 207.58) than from urine (Shannon index of 256.04 and Simpson diversity of 170.31; Supplementary Table S2).

### Peak Co-occurring Study

**Figure 7:**
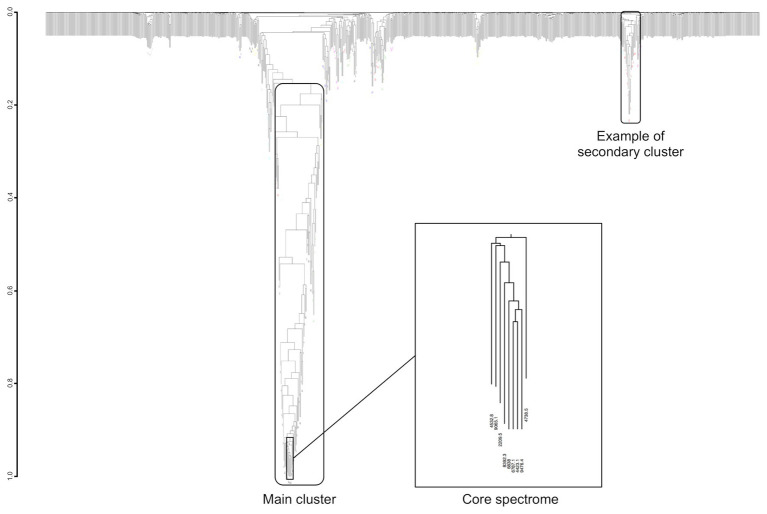
Example of dendrogram built from the MS peaks co-occurrence for *Streptococcus constellatus*. Its core spectrome of nine peaks captures 24% of main cluster ion current.

Co-occurring peak dendrograms are characterized by the presence of a main cluster of the most frequently co-occurring peaks, with several small secondary clusters, which gather less frequently associated peaks, and a large number of isolated peaks (Figure 7). We used the dynamic hybrid cut method for identifying the clusters, then their cores, which are the tip and most tightly connected peaks of each cluster (Langfelder et al., 2008).

From the 229 species having at least 10 spectra, we found an average of 25.48 clusters for each species (*SD* = 7.98, median = 25, first Qu. = 19, and third Qu. = 31). The average size of main clusters is 49.98 peaks (*SD* = 15.96, median = 49, first Qu. = 39, and third Qu. = 59), while their cores are made of an average of 7.52 peaks.

**Figure 8:**
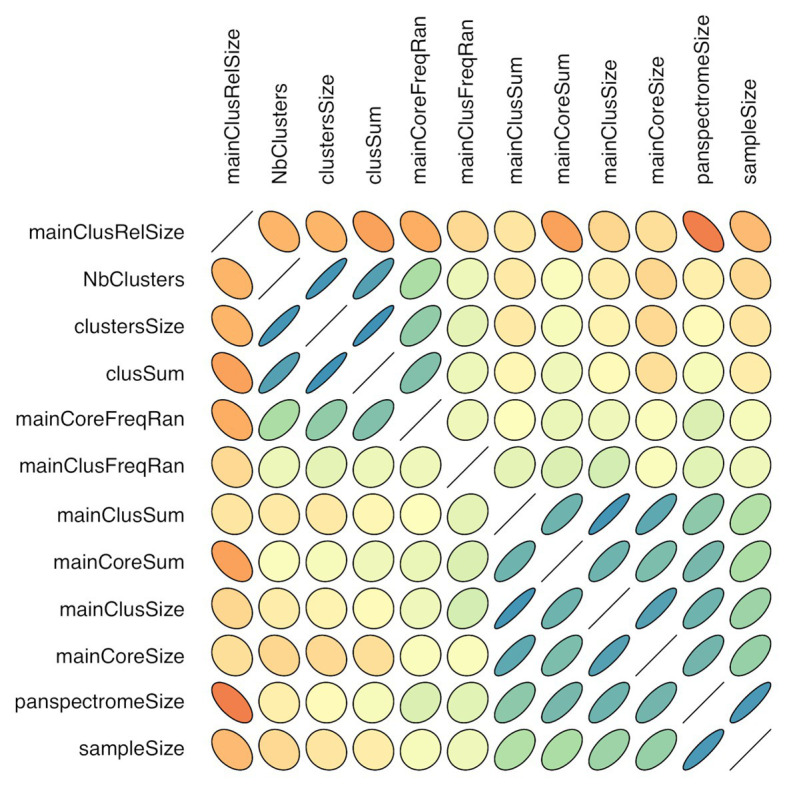
Correlogram of cluster characteristics (*mainClusRelSize*: relative size of the main cluster; *NbClusters*: number of clusters; *clustersSize*: number of peaks included in clusters; *clusSum*: ion current sum of peaks included in clusters; *mainCoreFreqRan*: frequency range between the most and least frequent core peak; *mainClusFreqRan*: frequency range between the most and least frequent main cluster peak; *mainClusSum*: ion current sum of main cluster peaks; *mainCoreSum*: ion current sum of core peaks; *mainClusSize*: number of main cluster peaks; *mainCoreSize*: number of core peaks; *panspectromeSize*: number of distinct species peaks; *SampleSize*: number of included spectra). Orientation of the ellipse depends on the correlation sign, shape and color depend on the correlation value (flat shape and intense color with increasing value).

There is a link between the sizes of the panspectrome, the main cluster and its core (Figure 8). A structural change study of the relation between the panspectrome and the species sample sizes using Bai and Perron’s method showed a change point of the curve at 651 spectra, suggesting that approximatively this number of spectra is required for a minimal species spectrome covering (Figure 5). Hence, clusters of only 35 bacterial species, corresponding to sample sizes over 600 spectra, were studied.

A database describing the cluster compositions of the 229 species (5,836 clusters) is available at the address https://www.mediterranee-infection.com/acces-ressources/base-de-donnees/.

The following of this study will focus only on these 35 best represented bacterial species.

#### Main Clusters

**Figure 9:**
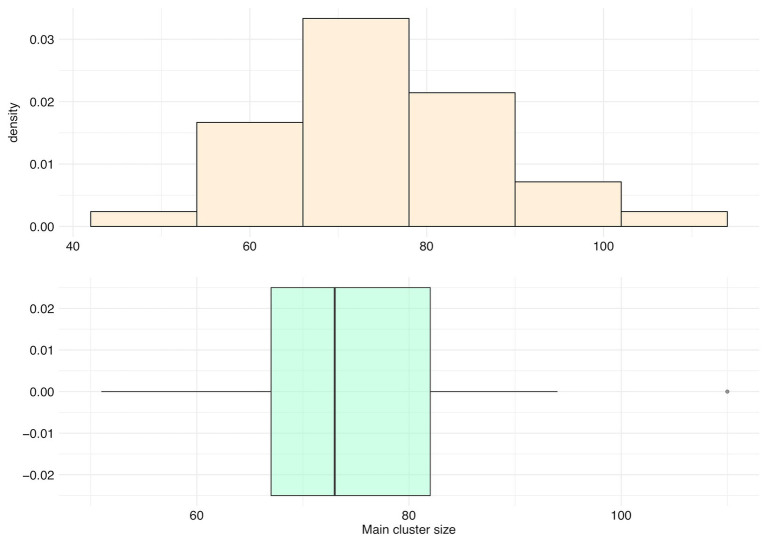
Distribution of the number of peaks included in the main cluster (main cluster size) for 35 species with a sample size over 600 spectra.

For all species, main clusters represent an average of only 5.80% of their panspectrome (*SD* = 1.71%, median = 5.68%, first Qu. = 4.55%, and third Qu. = 6.77%). The distribution of the number of peaks in main clusters is asymmetric, with a mean of 75.06 peaks (median = 73, first Qu. = 67, and third Qu. = 82; Figure 9). *Staphylococcus aureus* had the largest main cluster (110 peaks) and inversely *Corynebacterium striatum* the smallest (51 peaks).

Most of main cluster peaks have a frequency over 50% (Supplementary Figure S3). They represent an average of 51.6% ± 10.0% of clusters’ total ion current (median = 52.2%, first Qu. = 44.2%, and third Qu. = 57.9%). For example, *Streptococcus constellatus* has a main cluster of 70 peaks with a frequency varying between 13.6 and 97.9%, i.e., a frequency variation of 84.2% between the most and least frequent peak (Figure 7). Its main cluster captures 42% of clusters’ total ion current.

**Figure 10:**
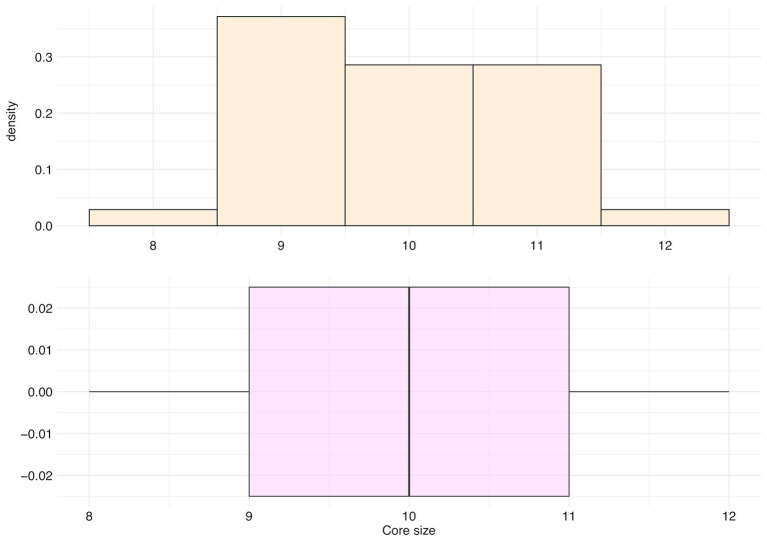
Distribution of the number of peaks belonging to the main cluster core (core size) for the top 35 species.

The main cluster cores of these 35 species were made of peaks with an occurrence frequency over 95%, and an average size of 9.91 peaks (*SD* = 0.95, median = 10 peaks, first Qu. = 9, and third Qu. = 11; Figure 10).

**Figure 11:**
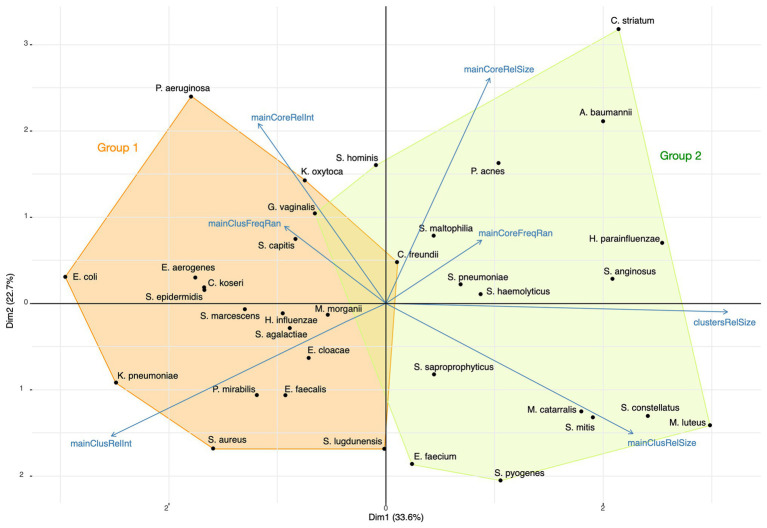
Principal Component Analysis (PCA) of the top 35 bacterial species with the composition of the two species groups identified by k-means (*mainCoreRelInt*: proportion of ions captured by the core; *mainClusFreqRan*: frequency range between the most and least frequent main cluster peak; *mainClusRelInt*: proportion of ions captured by the main cluster; *mainClusRelSize*: relative size of the main cluster; *clustersRelSize*: relative size of clusters; *mainCoreFreqRan*: frequency range between the most and least frequent core peak; *mainCoreRelSize*: relative size of the core).

Using a principal component analysis, we analyzed the relations between the relative sizes of the main cluster and its core, the proportion of ions captured and the peak relative frequency ranges for the 35 most represented species. The representation of this analysis on the two first dimensions is presented in Figure 11. K-means was used for finding the composition of species groups and a linear discriminant analysis (LDA) for confirming the grouping accuracy. The main axis (33.6% of the initial total variance) contrasts the proportion of ions captured by main clusters and their relative sizes, and the second axis (22.7% of the initial total variance) mainly the opposition between core and main cluster characteristics. The first group of bacterial species has its main clusters made of few proteins that concentrate a high total of ion current, while the second group has its main clusters made of a larger number of proteins associated with a broad distribution of the ion current over the protein peaks, showing a wider protein expression. These groups, identified by the k-means analysis, were confirmed by the LDA, with an accuracy of 0.97. Actually, a large main cluster do not imply a large core spectrome (Figure 11).

We have checked the presence of the six species peaks known to be systematically present in *Es. coli*, which were all retrieved in the main cluster of this species (RS32 [M + H]^+^, RS34 [M + H]^+^, and RS33 meth [M + H]^+^).

#### Secondary Clusters

The number of secondary clusters varied slightly between our 35 best represented bacterial species. There is in average 21.14 secondary clusters (*SD* = 4.78, median = 21, first Qu. = 18, and third Qu. = 25). Most of these peaks presented a relatively low occurrence frequency with a median of 20% (first Qu. = 11% and third Qu. = 34%: Supplementary Figure S4). These clusters captured an average of 48.4% of clusters’ total ion current (*SD* = 10%, first Qu. = 42.1%, and third Qu. = 55.8%). A Bai and Perron’s structural analysis of the distribution of number of clusters according to the sample sizes showed that 1,500 species spectra allow an overall convergence to an average of 20 secondary clusters for a bacterial species. This means that the acquisition cost of the secondary clusters of a panspectrome is around 1,500 spectra.

## Discussion

MALDI-TOF MS protein peaks are routinely used to identify bacteria at species level. The first way to do that was based on the retrieval of MS peaks corresponding to previously identified species characteristic proteins (Demirev et al., 1999). However, due to the difficulty to build a peak reference database and the lack of a constant specific peak expression, it has been dropped for an alternative method based on pattern matching (Freiwald and Sauer, 2009). This last method relies on the fact that spectra coming from a same species are similar, even if we do not know their protein peak composition. Then, like a fingerprinting, the MS spectrum of candidate bacteria is compared against a database gathering reference species spectra. The best similarity score (with a minimal threshold) gives the species identity. Our results show that even our 35 best represented species have no peak present in every spectrum, even if each of these species have a little set of peak presents in near 100% of them. This lack of species-specific peak constant expression may explain why the identification based on protein biomarkers is difficult in practice and why the similarity-based method is a more reliable and reproducible method, the absence of some peaks having few consequences on the identification. As main cluster peaks are the most frequently co-occurring species peaks, we may think that they cover the species peaks used in pattern matching.

Beside these species-specific peaks, the analysis of spectra showed the existence of secondary clusters made of frequently co-occuring peaks. This observation is in accordance with the possibility to do typing at an infra-species level, in the search for some subspecies specific peaks. Some examples exist for *Es. coli* (Matsumura et al., 2014; Novais et al., 2014), *Enterococcus faecium* (Griffin et al., 2012), *Haemophilus influenzae* (Månsson et al., 2015), or *S. aureus* (Josten et al., 2013; Ueda et al., 2015; Sauget et al., 2016). However, some other study results led to different conclusions on the ability of MALDI-TOF MS to discriminate bacterial subgroups. Sousa et al. (2015) did not result in the discrimination between various ST of *A. baumannii*, even in using chemometric methods. Our results suggest that sub-species typing must rely on the finding of clusters of co-occurring peaks and not of isolated peaks. We also showed that the sample size has an impact on the capability to identify secondary clusters, and that a minimum of about 600 spectra may be needed.

The spectra richness we have found, with an average of 101.02 peaks/spectrum is congruent with the known level of 100 peaks (Spinali et al., 2015). However, we have found more possible species-specific peaks (mean of 75.06 peaks) than the average of 54 peaks previously found (Spinali et al., 2015). This last discrepancy may be explained by the importance of our sample sizes (known as influencing peak diversity).

Using the peak statistic calculation tool included in the ClinProTools software directly on 51 strains, Nakamura et al. (2015) identified two protein peaks (7,650 and 7,707 Da) having the capability to distinguish *Es. coli* B2-ST131 from other ST clonal groups. We found these same co-occuring peaks in our spectra but with a variation of about 5 Da (respectively 7655.0 and 7702.5 Da) in 4,348 spectra (30.1%). This 600 ppm variability between the two studies can be explained by the 300 ppm tolerance during spectra acquisition, the spectra analysis method and the number of spectra included. In our study, we have tried to control a part of the well-known MALDI-TOF reproducibility problems by following workflow of Gibb and Strimmer (2012), and supplementing it with an additional target plate alignment step.

The principal component analysis of our 35 best represented species suggests that these species are distributed in two groups. These groups cannot be explained by the growing conditions because species growing on the two media are present in each group. The first group is characterized by a protein synthesis activity during the log phase that is concentrated on a small list of structural proteins, and the second group has a broader synthesis activity and a larger protein diversity. This last group gathers species like *Streptococci* that are the most difficult to identify by their spectra (Marín et al., 2017). We hypothesize that the more important expression of secondary clusters characterizing this group generates regular but not systematic patterns hindering MALDI-TOF species identification. In this case, a better identification would be possible with a focusing of the identification process on main cluster peaks only. A further work is required for exploring this unexpected observation.

This study describes some characteristics of species panspectromes, which is only a part of their panproteomes, as the acquisition of the MALDI-TOF spectra is done within a range from 2 to 20 kDa. MALDI-TOF MS spectra do not expose the protein expression of a strain in an exhaustive and global way. Their primary objective is only to display a standardized composition including at least peaks allowing the identification of the species. Medium and cell age are known to influence subspecies differentiation capabilities of MALDI-TOF MS (Ruelle et al., 2004), but perhaps differently according to species, as this influence has been reported (Freiwald and Sauer, 2009) or not (Seibold et al., 2010) by different works. However, the spectra we have used are coming from routine identification, with highly standardized protocol, and produced in such a way that a species is always coming from the same medium (optimal growing medium among two possibilities) and analyzed during the beginning of its log phase. We have also tried to control the automata-dependant drift with a target plate correction of spectra. Proteins carrying information with clinical or epidemiological impact may be located outside MALDI-TOF range and out of the scope of this study. MALDI-TOF MS Microflex®, as used in routine identification, is optimal between 3 and 15 kDa, raising the problem of the relevance of protein peaks detected outside this range. Precedent works show that most of discriminations at sub-species level are done with protein peaks not exceeding 10 kDa (Nakamura et al., 2015; Li et al., 2018), and more precisely between 4 and 10 kDa for Sousa et al. (2015) or between 2 and 9 kDa for Ueda et al. (2015). In addition, a peak is not always equivalent to a protein: a same protein peak may be due to the ionization of several proteins with the same mass to charge ratio and conversely, one protein may correspond to several peaks on a spectrum depending on its electrical charge.

Knowledge of the diversity of protein peaks shows a broad size dependent variability. The uncommon size of our MALDI-TOF spectra database allowed us to mine the composition of routine identification spectra from several species, and we have published the cluster composition of 229 species (5,836 clusters) at the address https://www.mediterranee-infection.com/acces-ressources/base-de-donnees/. Our results may advocate some analogy with genomics studies of bacteria, which showed that species characterization is brought by a core genome and strain characterization by the dispensable genes (Georgiades and Raoult, 2011; Caputo et al., 2019). For all species we have found that their panspectromes exhibit a structuring made of a main cluster of co-occurring peaks, which may be species specific, associated with several (around 20 by species) inconstant secondary clusters, which may host peaks able to discriminate bacterial subgroups. This diversity analysis was not intended to be used routinely, but its aim was to acquire findings valuable for the improving of routine processes. Current routine identification is based on spectra matching against reference databases and not on species-specific peak constant expression, which we have shown the non-existence. However, even this pattern matching has some difficulties with several species (Florio et al., 2018), including *Streptococci*. As suggested by our results, focusing pattern matching on main cluster or main cluster core peaks, excluding peaks from secondary clusters, would improve the reliability of species identifications. General spectra organization on clusters of co-occurring peaks lets suppose that subgroup typing or investigation of associations with clinical or epidemiological characteristics must rely on the finding of clusters of co-occurring peaks and not of isolated peaks. This would allow faster diagnosis, patient-oriented treatment and public health investigations.

## Data Availability Statement

The datasets presented in this study can be found in online repositories. The names of the repository/repositories and accession number(s) can be found at: https://www.mediterranee-infection.com/acces-ressources/donnees-pour-articles/maldi-tof-raw-data-spectrum-results/.

## Author Contributions

HC performed project design. AG-G, GT, and HC performed research, analysis, and paper drafting. DR performed critical revision for important intellectual content. All authors contributed to the article and approved the submitted version.

### Conflict of Interest

The authors declare that the research was conducted in the absence of any commercial or financial relationships that could be construed as a potential conflict of interest.
