# Supplementary material for: Insights Into Subspecies Discrimination Potentiality From Bacteria MALDI-TOF Mass Spectra by Using Data Mining and Diversity Studies
Source: Front Microbiol. 2020 Aug 13;11:1931. doi: 10.3389/fmicb.2020.01931 (PMC7438549; doi:10.3389/fmicb.2020.01931)
Supplement: Supplementary file 1 [file Data_Sheet_1.docx]

# Supplementary Table S1 – Distribution of panspectrome richness according to the number of included spectra and species.

| **Species** | **Number of included spectra** | **Panspectrome richness** |
| --- | --- | --- |
| *Staphylococcus epidermidis* | 14551 | 2724 |
| *Klebsiella pneumoniae* | 14455 | 2651 |
| *Escherichia coli* | 14450 | 2521 |
| *Staphylococcus aureus* | 14510 | 2469 |
| *Pseudomonas aeruginosa* | 14463 | 2439 |
| *Enterobacter cloacae* | 10533 | 2267 |
| *Enterococcus faecalis* | 10298 | 2211 |
| *Streptococcus agalactiae* | 5490 | 2178 |
| *Gardnerella vaginalis* | 7518 | 2158 |
| *Staphylococcus hominis* | 3093 | 1751 |
| *Staphylococcus capitis* | 1856 | 1692 |
| *Klebsiella oxytoca* | 2678 | 1630 |
| *Proteus mirabilis* | 5837 | 1600 |
| *Enterobacter aerogenes* | 2816 | 1583 |
| *Haemophilus influenzae* | 3945 | 1570 |
| *Staphylococcus haemolyticus* | 2361 | 1485 |
| *Propionibacterium acnes* | 1571 | 1482 |
| *Serratia marcescens* | 2578 | 1474 |
| *Staphylococcus lugdunensis* | 1414 | 1424 |
| *Staphylococcus saprophyticus* | 1171 | 1340 |
| *Citrobacter koseri* | 2014 | 1330 |
| *Enterococcus faecium* | 2143 | 1327 |
| *Streptococcus pneumoniae* | 1561 | 1282 |
| *Morganella morganii* | 1643 | 1271 |
| *Stenotrophomonas maltophilia* | 1261 | 1218 |
| *Corynebacterium striatum* | 735 | 1162 |
| *Streptococcus pyogenes* | 1074 | 1158 |
| *Haemophilus parainfluenzae* | 1007 | 1152 |
| *Acinetobacter baumannii* | 695 | 1144 |
| *Citrobacter freundii* | 1062 | 1130 |
| *Streptococcus anginosus* | 903 | 1049 |
| *Clostridium difficile* | 577 | 1036 |
| *Streptococcus mitis* | 657 | 1022 |
| *Streptococcus constellatus* | 814 | 999 |
| *Acinetobacter pittii* | 385 | 978 |
| *Micrococcus luteus* | 651 | 950 |
| *Achromobacter xylosoxidans* | 446 | 945 |
| *Streptococcus oralis* | 460 | 904 |
| *Moraxella catarrhalis* | 751 | 874 |
| *Streptococcus gallolyticus* | 317 | 807 |
| *Streptococcus dysgalactiae* | 306 | 790 |
| *Staphylococcus pasteuri* | 206 | 780 |
| *Raoultella ornithinolytica* | 155 | 780 |
| *Staphylococcus warneri* | 269 | 748 |
| *Klebsiella variicola* | 314 | 742 |
| *Bacteroides fragilis* | 384 | 741 |
| *Proteus vulgaris* | 265 | 700 |
| *Turicella otitidis* | 113 | 676 |
| *Staphylococcus caprae* | 205 | 670 |
| *Bacillus cereus* | 174 | 669 |
| *Hafnia alvei* | 322 | 668 |
| *Corynebacterium amycolatum* | 208 | 665 |
| *Neisseria gonorrhoeae* | 207 | 665 |
| *Rothia mucilaginosa* | 88 | 651 |
| *Staphylococcus simulans* | 189 | 649 |
| *Pseudomonas putida* | 116 | 647 |
| *Haemophilus haemolyticus* | 173 | 644 |
| *Aerococcus urinae* | 197 | 636 |
| *Streptococcus intermedius* | 231 | 624 |
| *Burkholderia multivorans* | 144 | 618 |
| *Corynebacterium urealyticum* | 112 | 616 |
| *Pasteurella multocida* | 270 | 605 |
| *Lactobacillus gasseri* | 219 | 601 |
| *Burkholderia cenocepacia* | 137 | 597 |
| *Capnocytophaga sputigena* | 230 | 589 |
| *Corynebacterium tuberculostearicum* | 102 | 587 |
| *Corynebacterium jeikeium* | 77 | 579 |
| *Haemophilus parahaemolyticus* | 165 | 578 |
| *Lactobacillus jensenii* | 170 | 576 |
| *Enterobacter asburiae* | 112 | 572 |
| *Finegoldia magna* | 96 | 565 |
| *Actinomyces turicensis* | 65 | 560 |
| *Staphylococcus pettenkoferi* | 141 | 556 |
| *Lactobacillus rhamnosus* | 103 | 553 |
| *Parvimonas micra* | 87 | 550 |
| *Citrobacter braakii* | 98 | 543 |
| *Streptococcus sanguinis* | 141 | 537 |
| *Staphylococcus schleiferi* | 96 | 528 |
| *Actinomyces neuii* | 60 | 525 |
| *Streptococcus salivarius* | 108 | 501 |
| *Actinomyces odontolyticus* | 68 | 499 |
| *Neisseria meningitidis* | 96 | 497 |
| *Aeromonas caviae* | 49 | 497 |
| *Campylobacter jejuni* | 159 | 494 |
| *Streptococcus gordonii* | 94 | 488 |
| *Aeromonas hydrophila* | 61 | 485 |
| *Lactobacillus iners* | 215 | 484 |
| *Lactobacillus delbrueckii* | 77 | 480 |
| *Corynebacterium propinquum* | 75 | 475 |
| *Providencia stuartii* | 100 | 473 |
| *Providencia rettgeri* | 96 | 471 |
| *Propionibacterium avidum* | 56 | 470 |
| *Enterobacter cancerogenus* | 76 | 467 |
| *Acinetobacter ursingii* | 56 | 466 |
| *Enterobacter kobei* | 79 | 463 |
| *Enterococcus avium* | 59 | 463 |
| *Dermabacter hominis* | 41 | 457 |
| *Staphylococcus cohnii* | 61 | 456 |
| *Corynebacterium simulans* | 53 | 452 |
| *Pantoea agglomerans* | 70 | 450 |
| *Acinetobacter lwoffii* | 70 | 449 |
| *Streptococcus parasanguinis* | 70 | 449 |
| *Acinetobacter nosocomialis* | 53 | 449 |
| *Kocuria rhizophila* | 38 | 449 |
| *Bacteroides thetaiotaomicron* | 91 | 448 |
| *Peptoniphilus harei* | 64 | 440 |
| *Corynebacterium glucuronolyticum* | 43 | 433 |
| *Corynebacterium pseudodiphtheriticum* | 74 | 428 |
| *Enterobacter ludwigii* | 46 | 428 |
| *Pseudomonas oryzihabitans* | 57 | 426 |
| *Neisseria flavescens* | 75 | 425 |
| *Eikenella corrodens* | 52 | 423 |
| *Fusobacterium nucleatum* | 59 | 418 |
| *Aeromonas veronii* | 42 | 415 |
| *Pantoea septica* | 47 | 414 |
| *Pseudomonas mosselii* | 51 | 410 |
| *Staphylococcus petrasii* | 33 | 408 |
| *Leclercia adecarboxylata* | 34 | 407 |
| *Corynebacterium aurimucosum* | 17 | 396 |
| *Escherichia hermannii* | 57 | 391 |
| *Granulicatella adiacens* | 30 | 390 |
| *Pseudomonas monteilii* | 52 | 386 |
| *Acinetobacter radioresistens* | 29 | 384 |
| *Actinomyces oris* | 34 | 376 |
| *Acinetobacter septicus* | 42 | 373 |
| *Clostridium perfringens* | 67 | 372 |
| *Pandoraea pulmonicola* | 71 | 365 |
| *Chryseobacterium gleum* | 36 | 360 |
| *Rothia dentocariosa* | 22 | 352 |
| *Staphylococcus xylosus* | 12 | 349 |
| *Staphylococcus pseudintermedius* | 60 | 348 |
| *Enterococcus casseliflavus* | 41 | 346 |
| *Moraxella osloensis* | 37 | 346 |
| *Actinotignum schaalii* | 38 | 342 |
| *Pseudomonas stutzeri* | 38 | 341 |
| *Staphylococcus saccharolyticus* | 29 | 340 |
| *Corynebacterium coyleae* | 19 | 339 |
| *Neisseria macacae* | 45 | 335 |
| *Enterococcus gallinarum* | 63 | 334 |
| *Corynebacterium accolens* | 20 | 333 |
| *Helicobacter pylori* | 17 | 332 |
| *Corynebacterium macginleyi* | 25 | 330 |
| *Moraxella nonliquefaciens* | 63 | 329 |
| *Chryseobacterium indologenes* | 14 | 328 |
| *Campylobacter fetus* | 51 | 327 |
| *Bacillus pumilus* | 36 | 326 |
| *Bacteroides ovatus* | 21 | 317 |
| *Clostridium tertium* | 19 | 316 |
| *Bifidobacterium scardovii* | 16 | 312 |
| *Pasteurella canis* | 54 | 311 |
| *Citrobacter amalonaticus* | 43 | 311 |
| *Fusobacterium necrophorum* | 38 | 311 |
| *Serratia liquefaciens* | 32 | 306 |
| *Lactobacillus crispatus* | 14 | 297 |
| *Aggregatibacter aphrophilus* | 28 | 295 |
| *Streptococcus australis* | 23 | 295 |
| *Alloscardovia omnicolens* | 16 | 294 |
| *Listeria monocytogenes* | 23 | 293 |
| *Burkholderia cepacia* | 20 | 293 |
| *Acinetobacter haemolyticus* | 14 | 293 |
| *Citrobacter sedlakii* | 22 | 289 |
| *Pantoea dispersa* | 39 | 284 |
| *Staphylococcus condimenti* | 11 | 284 |
| *Bacteroides vulgatus* | 34 | 283 |
| *Abiotrophia defectiva* | 39 | 274 |
| *Kocuria kristinae* | 16 | 274 |
| *Prevotella denticola* | 13 | 274 |
| *Serratia rubidaea* | 30 | 273 |
| *Staphylococcus piscifermentans* | 13 | 273 |
| *Streptococcus equinus* | 13 | 273 |
| *Nocardia farcinica* | 12 | 272 |
| *Corynebacterium minutissimum* | 9 | 271 |
| *Streptococcus massiliensis* | 23 | 270 |
| *Campylobacter coli* | 22 | 270 |
| *Bacillus megaterium* | 6 | 269 |
| *Acinetobacter junii* | 40 | 267 |
| *Actinomyces urogenitalis* | 20 | 265 |
| *Trueperella bernardiae* | 14 | 265 |
| *Enterococcus durans* | 16 | 262 |
| *Lactococcus lactis* | 30 | 260 |
| *Roseomonas mucosa* | 21 | 260 |
| *Pseudomonas plecoglossicida* | 20 | 260 |
| *Moraxella lacunata* | 20 | 259 |
| *Aerococcus sanguinicola* | 20 | 258 |
| *Streptococcus mutans* | 11 | 255 |
| *Gemella haemolysans* | 20 | 254 |
| *Pantoea calida* | 17 | 253 |
| *Achromobacter insolitus* | 8 | 253 |
| *Salmonella enterica* | 35 | 252 |
| *Brevibacterium casei* | 19 | 251 |
| *Prevotella bivia* | 30 | 248 |
| *Leuconostoc lactis* | 24 | 248 |
| *Staphylococcus auricularis* | 18 | 248 |
| *Clostridium sordellii* | 10 | 247 |
| *Lactobacillus casei* | 16 | 245 |
| *Prevotella melaninogenica* | 12 | 244 |
| *Lactobacillus paracasei* | 19 | 243 |
| *Lactococcus garvieae* | 26 | 241 |
| *Bacteroides cellulosilyticus* | 4 | 240 |
| *Lactobacillus salivarius* | 12 | 238 |
| *Prevotella massiliensis* | 1 | 238 |
| *Pseudomonas luteola* | 7 | 237 |
| *Lactobacillus johnsonii* | 14 | 236 |
| *Streptococcus vestibularis* | 14 | 236 |
| *Pasteurella stomatis* | 10 | 235 |
| *Janibacter sanguinis* | 4 | 235 |
| *Acinetobacter calcoaceticus* | 12 | 233 |
| *Corynebacterium durum* | 1 | 232 |
| *Delftia acidovorans* | 28 | 231 |
| *Actinomyces europaeus* | 9 | 231 |
| *Legionella pneumophila* | 12 | 230 |
| *Serratia odorifera* | 2 | 230 |
| *Proteus penneri* | 18 | 225 |
| *Corynebacterium kroppenstedtii* | 7 | 225 |
| *Aerococcus viridans* | 6 | 225 |
| *Yersinia enterocolitica* | 18 | 224 |
| *Pseudomonas mendocina* | 16 | 223 |
| *Raoultella planticola* | 16 | 223 |
| *Enterococcus hirae* | 14 | 223 |
| *Enterobacter hormaechei* | 4 | 223 |
| *Bacillus circulans* | 10 | 216 |
| *Actinomyces meyeri* | 8 | 215 |
| *Corynebacterium auris* | 4 | 215 |
| *Pseudomonas fulva* | 16 | 214 |
| *Proteus hauseri* | 22 | 213 |
| *Corynebacterium afermentans* | 8 | 213 |
| *Streptococcus pasteurianus* | 6 | 212 |
| *Pseudomonas massiliensis* | 4 | 211 |
| *Acinetobacter johnsonii* | 8 | 209 |
| *Clostridium clostridioforme* | 17 | 208 |
| *Parabacteroides distasonis* | 14 | 208 |
| *Streptococcus lutetiensis* | 12 | 207 |
| *Bacillus simplex* | 16 | 206 |
| *Alcaligenes faecalis* | 11 | 204 |
| *Bordetella bronchiseptica* | 10 | 204 |
| *Bifidobacterium breve* | 7 | 204 |
| *Comamonas kerstersii* | 6 | 204 |
| *Streptococcus minor* | 6 | 204 |
| *Streptococcus pseudopneumoniae* | 14 | 203 |
| *Aggregatibacter segnis* | 10 | 202 |
| *Fusobacterium naviforme* | 11 | 198 |
| *Nocardia abscessus* | 4 | 198 |
| *Clostridium bifermentans* | 4 | 197 |
| *Haemophilus pittmaniae* | 10 | 196 |
| *Pseudomonas fluorescens* | 6 | 196 |
| *Acinetobacter bereziniae* | 14 | 195 |
| *Bacteroides pyogenes* | 8 | 195 |
| *Dolosigranulum pigrum* | 8 | 194 |
| *Prevotella intermedia* | 6 | 193 |
| *Bacteroides uniformis* | 9 | 192 |
| *Propionibacterium granulosum* | 5 | 192 |
| *Burkholderia gladioli* | 10 | 190 |
| *Veillonella parvula* | 12 | 189 |
| *Actinomyces naeslundii* | 5 | 189 |
| *Neisseria mucosa* | 8 | 188 |
| *Enterococcus raffinosus* | 6 | 188 |
| *Corynebacterium imitans* | 4 | 188 |
| *Actinomyces radingae* | 8 | 186 |
| *Corynebacterium argentoratense* | 4 | 184 |
| *Brevundimonas diminuta* | 10 | 183 |
| *Escherichia vulneris* | 12 | 182 |
| *Prevotella oris* | 8 | 180 |
| *Dialister pneumosintes* | 4 | 180 |
| *Bacillus licheniformis* | 4 | 179 |
| *Pseudomonas koreensis* | 8 | 178 |
| *Elizabethkingia miricola* | 6 | 178 |
| *Atopobium rimae* | 4 | 177 |
| *Vibrio cholerae* | 4 | 176 |
| *Staphylococcus intermedius* | 6 | 175 |
| *Peptostreptococcus anaerobius* | 8 | 174 |
| *Elizabethkingia meningoseptica* | 16 | 173 |
| *Anaerococcus octavius* | 6 | 173 |
| *Gemella morbillorum* | 6 | 173 |
| *Streptococcus infantarius* | 3 | 173 |
| *Kluyvera ascorbata* | 12 | 172 |
| *Vibrio alginolyticus* | 6 | 172 |
| *Streptococcus cristatus* | 5 | 172 |
| *Atopobium vaginae* | 4 | 172 |
| *Leuconostoc mesenteroides* | 4 | 171 |
| *Acinetobacter schindleri* | 6 | 170 |
| *Neisseria subflava* | 12 | 169 |
| *Bacteroides caccae* | 6 | 169 |
| *Staphylococcus sciuri* | 4 | 168 |
| *Gemella sanguinis* | 2 | 168 |
| *Clostridium septicum* | 3 | 166 |
| *Haemophilus sputorum* | 10 | 163 |
| *Clostridium subterminale* | 2 | 163 |
| *Aeromonas media* | 4 | 161 |
| *Atopobium parvulum* | 8 | 160 |
| *Arcanobacterium haemolyticum* | 4 | 160 |
| *Bacillus thuringiensis* | 4 | 160 |
| *Prevotella nigrescens* | 6 | 157 |
| *Granulicatella elegans* | 2 | 157 |
| *Facklamia languida* | 3 | 156 |
| *Capnocytophaga gingivalis* | 6 | 155 |
| *Staphylococcus gallinarum* | 4 | 154 |
| *Nocardia nova* | 2 | 153 |
| *Sphingomonas paucimobilis* | 8 | 152 |
| *Anaerococcus vaginalis* | 4 | 152 |
| *Brevibacillus agri* | 2 | 150 |
| *Corynebacterium mucifaciens* | 2 | 150 |
| *Microbacterium oxydans* | 2 | 150 |
| *Paenibacillus pabuli* | 4 | 149 |
| *Staphylococcus arlettae* | 2 | 149 |
| *Lactobacillus fermentum* | 4 | 147 |
| *Bifidobacterium longum* | 3 | 147 |
| *Staphylococcus equorum* | 3 | 146 |
| *Corynebacterium bovis* | 2 | 146 |
| *Alistipes finegoldii* | 4 | 145 |
| *Citrobacter farmeri* | 8 | 143 |
| *Microbacterium lacticum* | 2 | 142 |
| *Rothia terrae* | 2 | 142 |
| *Leptotrichia trevisanii* | 6 | 141 |
| *Slackia exigua* | 3 | 140 |
| *Corynebacterium pseudotuberculosis* | 2 | 140 |
| *Paenibacillus provencensis* | 2 | 140 |
| *Ochrobactrum intermedium* | 7 | 139 |
| *Bacillus amyloliquefaciens* | 1 | 137 |
| *Veillonella atypica* | 6 | 136 |
| *Neisseria cinerea* | 4 | 136 |
| *Streptococcus urinalis* | 4 | 136 |
| *Brevibacterium luteolum* | 4 | 135 |
| *Cronobacter sakazakii* | 4 | 135 |
| *Streptococcus peroris* | 2 | 135 |
| *Pasteurella dagmatis* | 4 | 134 |
| *Microbacterium paraoxydans* | 2 | 133 |
| *Kingella kingae* | 4 | 132 |
| *Kytococcus schroeteri* | 2 | 131 |
| *Lactobacillus sakei* | 2 | 131 |
| *Eubacterium limosum* | 4 | 130 |
| *Corynebacterium lascolaensis* | 2 | 130 |
| *Serratia ficaria* | 4 | 128 |
| *Actinobaculum massiliense* | 2 | 128 |
| *Listeria ivanovii* | 2 | 128 |
| *Lysinibacillus fusiformis* | 1 | 127 |
| *Prevotella buccae* | 6 | 126 |
| *Kocuria palustris* | 2 | 126 |
| *Lactobacillus vaginalis* | 2 | 126 |
| *Clostridium paraputrificum* | 4 | 125 |
| *Lactobacillus reuteri* | 4 | 125 |
| *Neisseria lactamica* | 4 | 125 |
| *Bacillus clausii* | 2 | 125 |
| *Clostridium baratii* | 4 | 124 |
| *Actinomyces ihumii* | 2 | 124 |
| *Fusobacterium gonidiaformans* | 4 | 123 |
| *Actinomyces israelii* | 2 | 123 |
| *Eggerthella lenta* | 2 | 123 |
| *Raoultella terrigena* | 2 | 122 |
| *Campylobacter rectus* | 1 | 122 |
| *Bacillus weihenstephanensis* | 4 | 121 |
| *Moraxella lincolnii* | 2 | 120 |
| *Streptococcus canis* | 2 | 119 |
| *Bacillus badius* | 4 | 118 |
| *Bacillus firmus* | 2 | 118 |
| *Ochrobactrum anthropi* | 4 | 117 |
| *Streptococcus castoreus* | 2 | 117 |
| *Corynebacterium diphtheriae* | 1 | 117 |
| *Brevundimonas aurantiaca* | 4 | 116 |
| *Kluyvera georgiana* | 2 | 115 |
| *Prevotella conceptionensis* | 2 | 115 |
| *Oligella urethralis* | 4 | 114 |
| *Akkermansia muciniphila* | 2 | 114 |
| *Prevotella disiens* | 2 | 114 |
| *Acidovorax temperans* | 2 | 113 |
| *Clostridium cadaveris* | 2 | 113 |
| *Pseudomonas alcaligenes* | 2 | 113 |
| *Acinetobacter parvus* | 2 | 112 |
| *Paracoccus yeei* | 2 | 112 |
| *Porphyromonas gingivalis* | 2 | 110 |
| *Delftia tsuruhatensis* | 4 | 109 |
| *Corynebacterium confusum* | 2 | 108 |
| *Roseomonas genomospecies 5* | 4 | 107 |
| *Lactobacillus plantarum* | 1 | 106 |
| *Cupriavidus gilardii* | 6 | 105 |
| *Neisseria elongata* | 2 | 105 |
| *Cupriavidus respiraculi* | 1 | 105 |
| *Anaerotruncus colihominis* | 2 | 104 |
| *Bacillus marisflavi* | 2 | 104 |
| *Citrobacter youngae* | 2 | 104 |
| *Fusobacterium varium* | 2 | 104 |
| *Yersinia intermedia* | 2 | 104 |
| *Brevibacterium celere* | 2 | 101 |
| *Streptococcus equi* | 1 | 101 |
| *Citrobacter murliniae* | 2 | 99 |
| *Empedobacter brevis* | 2 | 98 |
| *Janibacter hoylei* | 1 | 98 |
| *Achromobacter denitrificans* | 4 | 96 |
| *Neisseria canis* | 2 | 95 |
| *Peptoniphilus grossensis* | 2 | 94 |
| *Bacillus mycoides* | 2 | 91 |
| *Vibrio vulnificus* | 2 | 89 |
| *Capnocytophaga ochracea* | 2 | 88 |
| *Comamonas testosteroni* | 2 | 88 |
| *Anaerococcus prevotii* | 1 | 87 |
| *Prevotella buccalis* | 2 | 86 |
| *Clostridium innocuum* | 2 | 85 |
| *Prevotella oulorum* | 2 | 84 |
| *Bacteroides stercoris* | 2 | 82 |
| *Vibrio parahaemolyticus* | 2 | 82 |
| *Ruminococcus gnavus* | 2 | 81 |
| *Bacillus subtilis* | 2 | 80 |
| *Peptoniphilus asaccharolyticus* | 2 | 74 |
| *Turicibacter sanguinis* | 2 | 71 |
| *Serratia ureilytica* | 1 | 70 |
| *Bordetella petrii* | 1 | 69 |

**Supplementary table S2 –** Diversity according to the kind of sample

|  | **Observed** | **Estimator** | **Est s.e.** | **.95 Lower** | **.95 Upper** |
| --- | --- | --- | --- | --- | --- |
| **Urine** |  |  |  |  |  |
| Species Richness | 2128 | 2217.550 | 19.407 | 2186.844 | 2264.278 |
| Shannon diversity | 256.036 | 256.431 | 0.346 | 256.036 | 257.110 |
| Simpson diversity | 170.310 | 170.326 | 0.167 | 170.310 | 170.653 |
| **Blood** |  |  |  |  |  |
| Species Richness | 2062 | 2223.314 | 27.387 | 2177.927 | 2286.472 |
| Shannon diversity | 344.314 | 346.330 | 0.896 | 344.574 | 348.086 |
| Simpson diversity | 207.584 | 207.690 | 0.446 | 207.584 | 208.565 |
| **Cardiac** |  |  |  |  |  |
| Species Richness | 416 | 657.131 | 55.983 | 569.893 | 793.820 |
| Shannon diversity | 224.922 | 245.729 | 4.113 | 237.668 | 253.790 |
| Simpson diversity | 169.988 | 173.559 | 2.005 | 169.988 | 177.488 |
| **Other** |  |  |  |  |  |
| Species Richness | 1954 | 2127.567 | 27.532 | 2081.434 | 2190.403 |
| Shannon diversity | 302.754 | 304.566 | 0.861 | 302.878 | 306.254 |
| Simpson diversity | 193.874 | 193.964 | 0.393 | 193.874 | 194.734 |


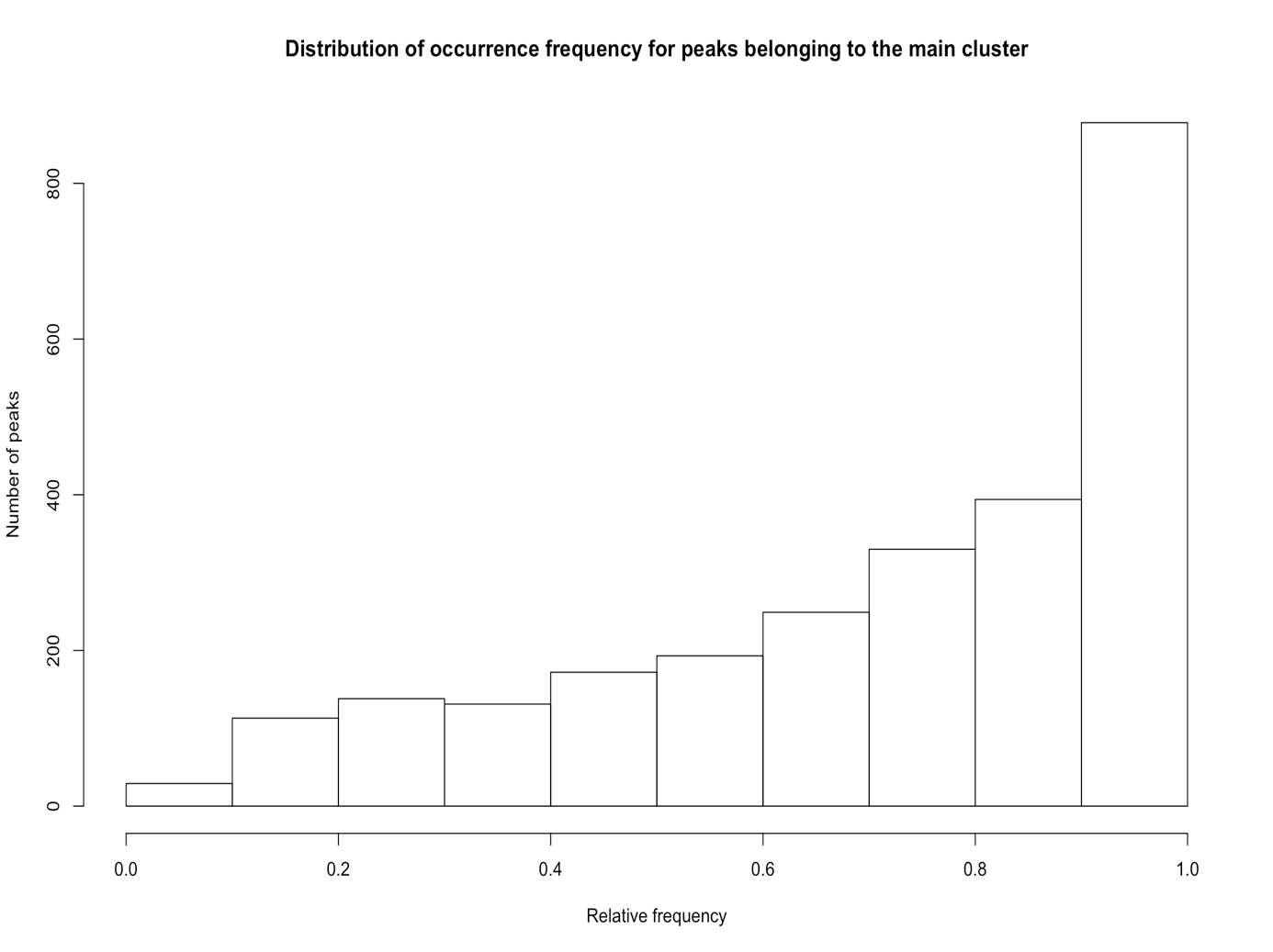


**Supplementary figure S3** – Frequency distribution of co-occurring peaks belonging to the main cluster. In average, these peaks are present in 0.71 of all spectra for a same bacterial species (median = 0.79, 1st Qu. = 0.54, 3rd Qu. = 0.94, min = 0.03, max =1.00)


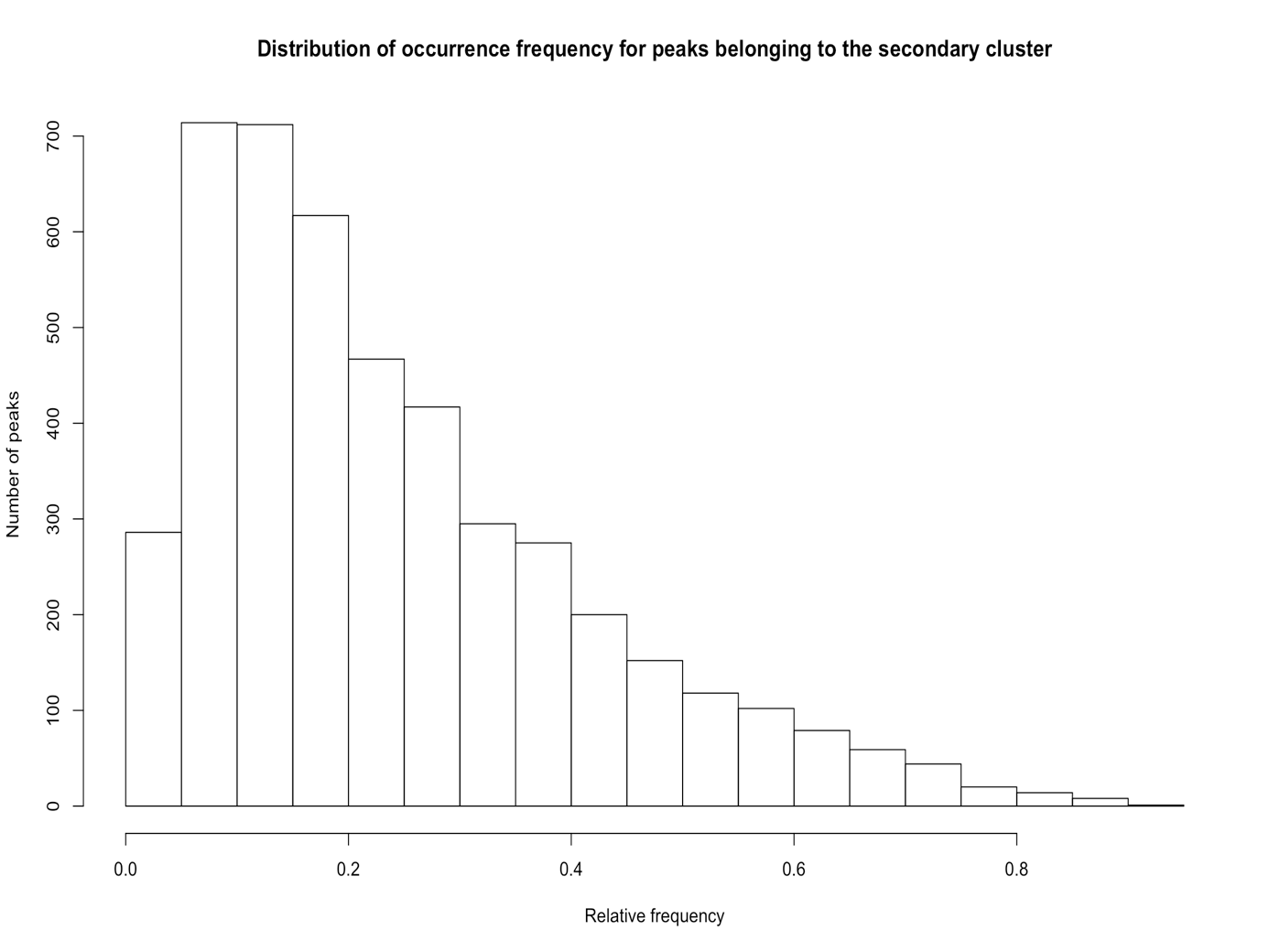


**Supplementary figure S4** – Frequency distribution of co-occurring peaks belonging to the secondary clusters. In average, these peaks are presented in 0.24 of all spectra for a same bacterial species (median = 0.120, 1st Qu. = 0.11, 3rd Qu. = 0.34, min = 0.01, max = 0.90).
